# Emerging sporotrichosis is driven by clonal and recombinant *Sporothrix* species

**DOI:** 10.1038/emi.2014.33

**Published:** 2014-05-07

**Authors:** Anderson Messias Rodrigues, GSybren de Hoog, Yu Zhang, Zoilo Pires de Camargo

**Affiliations:** 1 Department of Microbiology, Immunology and Parasitology, Cell Biology Division, Federal University of São Paulo, São Paulo 04023-062, Brazil; 2 Centraalbureau voor Schimmelcultures, KNAW Fungal Biodiversity Centre, Utrecht 3508 AD, The Netherlands

**Keywords:** emerging infectious diseases, epidemiology, fungi, outbreak, *Sporothrix*, sporotrichosis, zoonosis

## Abstract

Sporotrichosis, caused by agents of the fungal genus *Sporothrix*, occurs worldwide, but the infectious species are not evenly distributed. *Sporothrix* propagules usually gain entry into the warm-blooded host through minor trauma to the skin from contaminated plant debris or through scratches or bites from felines carrying the disease, generally in the form of outbreaks. Over the last decade, sporotrichosis has changed from a relatively obscure endemic infection to an epidemic zoonotic health problem. We evaluated the impact of the feline host on the epidemiology, spatial distribution, prevalence and genetic diversity of human sporotrichosis. Nuclear and mitochondrial markers revealed large structural genetic differences between *S. brasiliensis* and *S. schenckii* populations, suggesting that the interplay of host, pathogen and environment has a structuring effect on the diversity, frequency and distribution of *Sporothrix* species. Phylogenetic data support a recent habitat shift within *S. brasiliensis* from plant to cat that seems to have occurred in southeastern Brazil and is responsible for its emergence. A clonal structure was found in the early expansionary phase of the cat–human epidemic. However, the prevalent recombination structure in the plant-associated pathogen *S. schenckii* generates a diversity of genotypes that did not show any significant increase in frequency as etiological agents of human infection over time. These results suggest that closely related pathogens can follow different strategies in epidemics. Thus, species-specific types of transmission may require distinct public health strategies for disease control.

## INTRODUCTION

The fungal genus *Sporothrix*, which belongs to the plant-associated order Ophiostomatales, comprises a small group of ascomycetes, including a few clusters of species with a remarkable ability to cause infections in mammalian hosts. The four main pathogenic species diverge according to their geographical distribution, ecological niche and transmission routes. This divergence is reflected in species-specific arrays of prevalent hosts and habitats for which either plant material or felines are the main source of infection.

Sporotrichosis is a chronic infection of the skin and subcutaneous tissues. Benjamin Schenck first described the disease in 1898, and for more than a century, it was attributed to a sole etiological agent: *Sporothrix schenckii*. The thermodimorphic fungus grows as a mold in nature (25–30 °C) and converts to a yeast-like phase at elevated temperatures (35–37 °C), when propagules are traumatically introduced into the warm-blooded host. Infections range from fixed localized cutaneous lesions to severe disseminated sporotrichosis.^1,2,3^

The application of molecular tools has led to the description of four cryptic species recognized in clinical practice.^4,5^ The former species, *Sporothrix schenckii*, now comprises *S. brasiliensis* (clade I), *S. schenckii sensu stricto (s. str.)* (clade II), *S. globosa* (clade III) and *S. luriei* (clade VI).^6,7^ Pathogenicity to mammals is rarely observed outside this clade of four species, and only a few reports of infections by *S. mexicana* and^1^ *S. pallida*,^6,8^ or by close relatives in the genus *Ophiostoma*, i.e., *O. piceae*^9^ and *O. stenoceras*,^10^ have appeared in the literature.

Sporotrichosis has a worldwide occurrence, but the distinct etiological agents are not evenly distributed. Most endemic areas are located in somewhat warmer regions.^1,11,12^ A high prevalence has been reported in South Africa, India, Australia, China, Japan, the United States and Mexico. In South America, endemic areas include Uruguay, Peru, Colombia, Venezuela and Brazil. *Sporothrix* species are exceptional in the fungal kingdom due to their frequent occurrence in the form of outbreaks. With fundamental differences between the sources of outbreaks, host–environment interactions are classic determinants of risk factors for disease acquisition and public health measures must change accordingly.

In Brazil, isolated cases, small outbreaks and case series have been sporadically reported. However, over the last few decades, the southeastern part of the country has been experiencing a very large epidemic due to zoonotic transmission,^13^ with cats being the main vector through which the disease is transmitted to humans and other animals.^7,13,14^ Thousands of *Sporothrix* infections persist for many months in symptomatic and asymptomatic cats, leading to the transmission of sporotrichosis by cat-to-cat and cat-to-human contact patterns. The growing reservoir of infection causes continues to spread with epidemic proportions.^7^ The zoonotic transmission of *Sporothrix* through cats clearly differentiates Brazil from other outbreaks worldwide, where the source and vector of infection are primarily soil and decomposing organic matter. The predominant etiological agent in cats is *S. brasiliensis*,^7^ which is the most virulent species in the complex.^15,16^ Its occurrence is geographically restricted to the South and Southeast regions of Brazil.^1,5,6^

All species of the pathogenic cluster have been reported from Brazil,^1,7,12,17,18^ where habitat shifts between species are likely to occur. We investigated the recent, sudden emergence of sporotrichosis in South America. To assess trends in the epidemiology and genetic diversity of clinical isolates, we studied the Brazilian strains of *Sporothrix* that have been collected over a 60-year period. For comparison, we examined a collection of well-characterized isolates from the United States, Mexico, Peru, Japan, China, South Africa, United Kingdom, Italy and Spain.

## MATERIALS AND METHODS

### Fungal strains

A total of 204 isolates originally received as *S. schenckii* were included in this study. The isolates were of environmental, clinical or veterinary origin from different geographic regions of Brazil. For comparison, 28 reference isolates from outside the Brazilian territory were used. In addition, 75 calmodulin sequences belonging to Brazilian isolates were collected from GenBank. The total data set comprised 307 operational taxonomic units (Supplementary Table S1). Ethical approval was provided by Institutional Committee (UNIFESP-0244/11).

### Molecular characterization

Total DNA was obtained and purified directly from 10-day-old colonies on slants by following the Fast DNA kit protocol (MP Biomedicals, Vista, CA, USA).^1^ Polymerase chain reaction (PCR) amplification and DNA sequencing of specific regions of the calmodulin gene (*CAL*) and the rRNA operon^6^ were performed using the degenerate primers CL1 and CL2A^19^ and ITS1 and ITS4,^20^ respectively. To evaluate the genetic diversity and to assess mitochondrial haplotype differences among the isolates, a hypervariable intergenic region between the *ATP9* and *COX2* genes in the mitochondrial genome (mtDNA) was amplified and sequenced using primers 975–8038F and 975–9194R.^21^

The amplified products were gel-purified with the Wizard SV Gel and PCR Clean-Up System (Promega, Madison, WI, USA) following the manufacturer's instructions. The PCR products were sequenced directly in two reactions with forward and reverse primers to increase the quality of the sequence data (Phred ≥30). The sequencing reactions were carried out with the BigDye Terminator v3.1 Cycle Sequencing Kit (Applied Biosystems, Inc., Foster City, CA, USA), and the sequencing products were determined using an ABI 3730 DNA Analyzer 48-well capillary sequencer (Applied Biosystems, Inc., Foster City, CA, USA). The sequences generated in both orientations were assembled into single sequences via CAP3 implemented in BioEdit software. Sequences were aligned with MAFFT v.7,^22^ and retrieved alignments were manually edited to avoid mispaired bases. All sequences were deposited online at GenBank (Supplementary Table S1).

### Phylogenetic inference

Genetic relationships were investigated by phylogenetic analysis using neighbor-joining, maximum likelihood and maximum parsimony methods. Phylogenetic trees were constructed in MEGA5.^23^ Evolutionary distances were computed using the Kimura two-parameter,^24^ and the robustness of branches was assessed by a bootstrap analysis of 1000 replicates.^25^

### Haplotype network and recombination analysis

The nucleotide (*π*) and the haplotype (Hd) diversities^26^ were estimated using DnaSP software version 5.10.^27^ Sites containing gaps and missing data were not considered in the analysis. A haplotype network analysis was constructed using the Median-Joining method^28^ and implemented into the software NETWORK 4.6.1.0 (Fluxus-Technology). In addition, recombination possibilities were investigated using the NeighborNet method,^29^ which leads to reticulated relationships in the presence of recombination, as described by the Uncorrected-*P* distance or by the splits decomposition method,^30^ both implemented in the SplitsTree v.4b06.^31^ Additional measures of recombination were estimated using the PHI-test (*P*<0.05 demonstrated significant evidence of recombination).

## RESULTS

**Figure 1:**
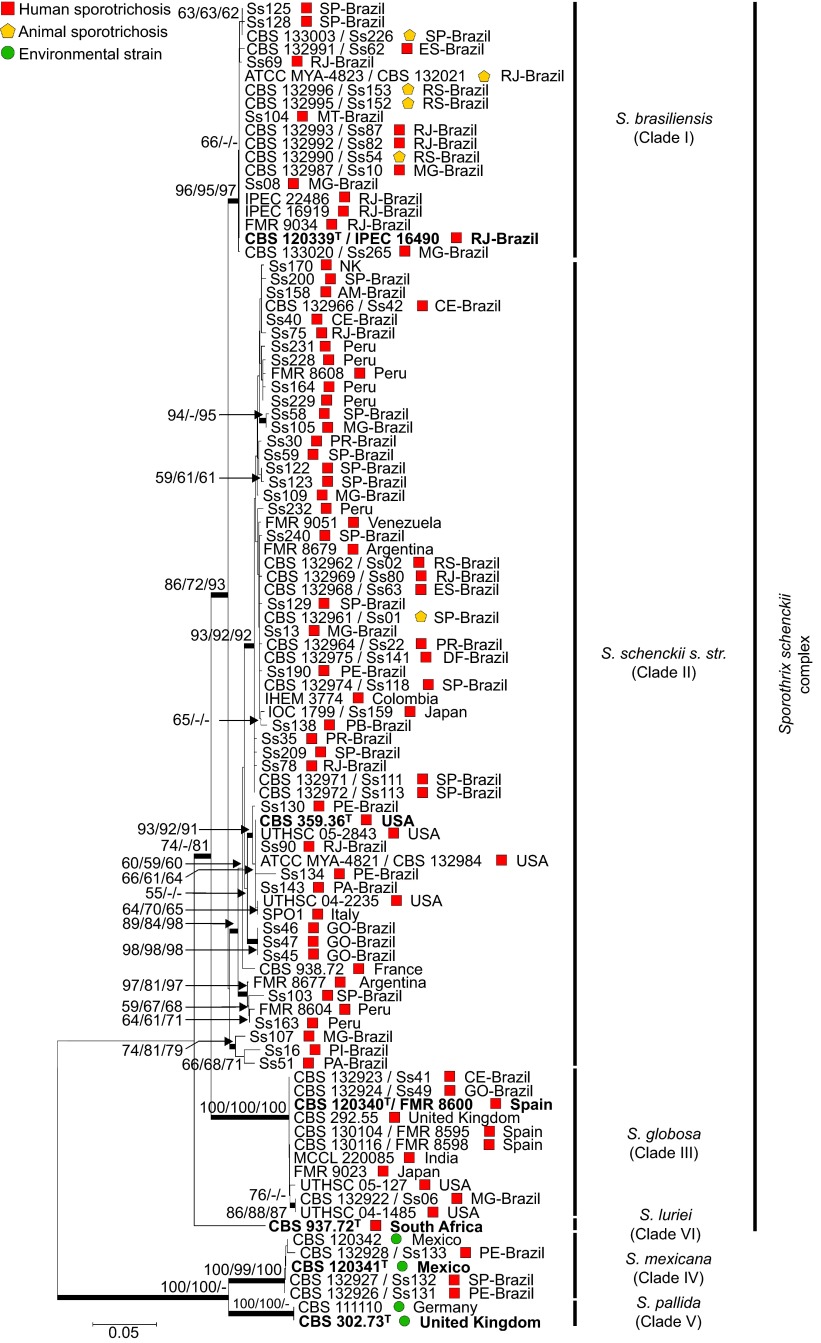
Phylogenetic relationships, as inferred from a maximum likelihood analysis of the *CAL* sequences from 98 strains, covering the main haplotypes of the *Sporothrix schenckii* complex circulating in Brazil. The numbers close to the branches represent indices of support (NJ/ML/MP) based on 1000 bootstrap replications. The branches with bootstrap support higher than 70% are indicated in bold. ML, maximum likelihood; MP, maximum parsimony; NJ, neighbor-joining. Further information about isolate source and GenBank accession number can be found in the Supplementary Table S1.

Based on the *CAL*, the data set for the molecular phylogeny of the *Sporothrix* isolates comprised 307 operational taxonomic units, as represented here by 98 specimens (Figure 1). The aligned *CAL* sequences were 710 bp long, including 409 invariable characters, 225 variable parsimony-informative sites (31.69%) and 52 singletons. The ITS sequences, including ITS1/2+5.8S regions were 634 bp long; of these, 375 characters were constant, 152 characters were variable parsimony-informative (23.97%) and 75 were singletons (Supplementary Figure S1). The phylogenies were concordant with high bootstrap support.

*Sporothrix brasiliensis* isolates were recovered from four out of five Brazilian regions and clustered together in a monophyletic clade (Clade I; 96/95/97) including human and animal isolates. *S. schenckii s. str*. was found to be more diverse, with several well-supported clusters in Brazil. The strains in clade II mostly originated from human cases of sporotrichosis, whereas the *S. brasiliensis* hosts were human (79.62%) and feline (20.37%). Very few Brazilian isolates of *S. globosa* have been reported in the literature, and the low incidence of this species is reflected in our data set. They form a well-supported clade (100/100/100) together with European, Asian, and North American strains of *S. globosa* (Figure 1).

**Figure 2:**
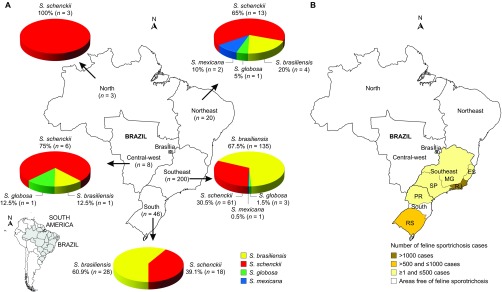
Distribution patterns of 277 *Sporothrix* spp. in Brazil. (**A**) Distribution of *Sporothrix* isolates in Brazil. (**B**) Distribution of feline sporotrichosis in Brazil, as based on outbreak reports in the literature.^7,13,32,33,34^ ES, Espírito Santo; MG, Minas Gerais; PR, Paraná RJ, Rio de Janeiro; RS, Rio Grande do Sul; SP, São Paulo.

The spatial trends in the Brazilian epidemic are shown in Figure 2. *S. brasiliensis* was found to be geographically restricted to the feline outbreak areas in the southeastern provinces of Brazil, whereas *S. schenckii s. str*. was found to be broadly distributed throughout Brazil. The Northeast and Southeast regions of the country presented four *Sporothrix* species (*S. brasiliensis*, *S. schenckii*, *S. globosa* and *S. mexicana*). Three species (*S. brasiliensis*, *S. schenckii* and *S. globosa*) were detected in the Central-West region. Only two species were detected (*S. brasiliensis* and *S. schenckii*) in the South region, whereas the only three strains in the North region were classified as *S. schenckii* (Figure 2A).

The influence of zoonotic transmission was undetected in the North, Northeast and Central-West regions (Figure 2B).^7,13,32,33,34^ Furthermore, the incidence of the disease among humans in these areas was lower than in the feline outbreak areas; a high incidence was observed in the South (*n*=46) and Southeast (*n*=200). These variances in incidence are supported by differences in the prevalent etiological agent. The high prevalence of *S. brasiliensis* in humans and animals overlaps geographically.

**Figure 3:**
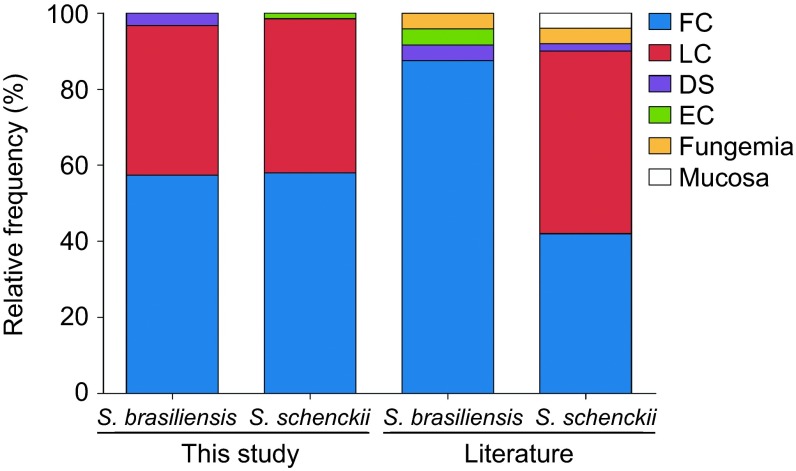
Frequency of clinical pictures between the two most common species in the *Sporothrix schenckii* complex. The frequencies are compared with previous data reported in the literature.^4,5,35^ DS, disseminated; EC, extracutaneous; fungemia and mucosa forms; FC, fixed cutaneous; LC, lymphocutaneous.

There were no differences observed in the clinical pictures among phylogenetic species. The 130 clinical isolates, which were genotyped by partial *CAL* sequences, were scattered among four clades. Some 57.3% and 57.9% of the isolates were from fixed cutaneous lesions, and 39.3% and 40.5% were from lymphocutaneous lesions of *S. brasiliensis* and *S. schenckii*, respectively (Figure 3).^4,5,35^ Taken together, 96.6%–98.4% of the clinical cases of human sporotrichosis in Brazil were of the fixed cutaneous and lymphocutaneous forms, independent of the etiological agent. In addition, 3.2% of the disseminated cases were *S. brasiliensis*, despite the lack of a severe case among the *S. schenckii* isolates.

**Figure 4:**
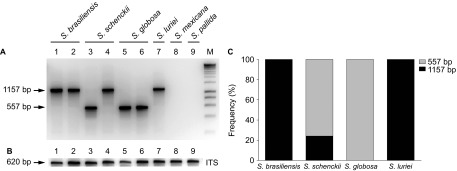
Mitochondrial genotyping of *Sporothrix* spp. isolates. (**A**) Genotyping results of polymorphic amplicons, which represent the variability of the intergenic region between *COX2* and *ATP9* in the mitochondrial genome of different isolates. (**B**) DNA quality control amplification of the nuclear rRNA operon. (**C**) Distribution of polymorphic amplicons, according to the phylogenetic species.

Genotyping of the Brazilian *Sporothrix* population was performed using an intergenic region in the mtDNA between the *ATP9* and *COX2* genes.^21^ Positive amplification was obtained from the pathogenic species in the clinical complex (Clades I, II, III and VI; Figure 4A) but not in the environmental clades (Clades IV and V), regardless of positive ITS amplification for DNA quality control (Figure 4B). All *S. brasiliensis* (*n*=82, 100%) strains that were genotyped presented amplicons of 1157 bp, identical to the amplicon found in the single strain (CBS 937.72) that was available for *S. luriei*. *Sporothrix schenckii* (*n*=87) was split into two groups: the major group (cluster 1, *n*=66, 75.86%) presented a single amplicon of 557 bp, whereas a smaller group (cluster 2, *n*=21, 24.13%) presented a single amplicon of 1157 bp (Figure 4C).

**Figure 5:**
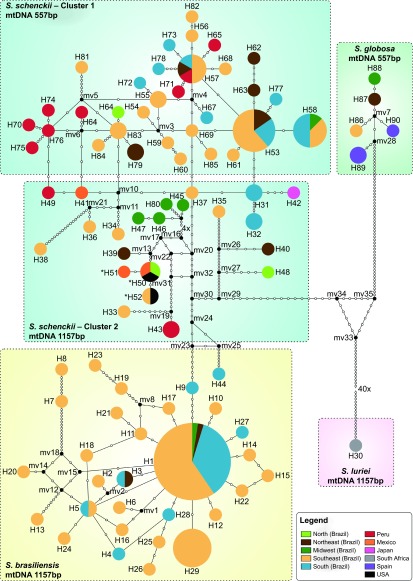
Median-joining haplotype network of *Sporothrix schenckii* complex isolates, covering all the concatenated mtDNA and *CAL* haplotypes found in this study. The size of the circumference is proportional to the haplotype frequency. The isolates are coded, and their frequencies are represented by geographic region of isolation. Mutational steps are represented by white dots. The black dots (median vectors) represent unsampled or extinct haplotypes in the population. Three haplotypes (H50–H52), indicated by a bold asterisk (*), present the 557 bp mtDNA rather than the major 1157 bp mtDNA of the group to which they are assigned.

The haplotype analysis of concatenated calmodulin and mtDNA sequences (*n*=176) divided the isolates into 90 Hap groups (Figure 5). A total of 30 and 54 different types were detected for *S. brasiliensis* and *S. schenckii s. str*., respectively. The majority of haplotypes (Hd=0.98) belonged to *S. schenckii*, constituting a highly diverse group (*π*=0.011).

The major group (cluster 1; 557 bp mtDNA) of *S. schenckii* isolates was retrieved from clinical cases and was related to the 1990s outbreak that originated in Brazil and Peru. The 1157 bp mtDNA group (cluster 2; *n*=21) dates back to the 1970s, with no significant increase in frequency as etiological agents of human infection over time. This group was geographically heterogeneous, as it was recovered from areas outside Brazil, including Peru, Mexico, Japan and the United States. The only exceptions in the 1157 bp mtDNA group are haplotypes H50–H52 (from Mexico, the United States and Brazil), which presented the 557 bp mtDNA amplicon but were otherwise genetically similar; thus, they may represent a link between the two groups or are hybrid isolates.

**Figure 6:**
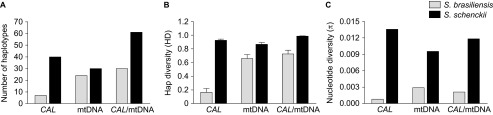
Genetic diversity in the *Sporothrix brasiliensis* and *S. schenckii* species, as explored by nuclear and mitochondrial markers. (**A**) Number of haplotypes, (**B**) haplotype and (**C**) nucleotide diversity among the etiological agents of sporotrichosis. The thin bars represent the standard deviation.

*S. brasiliensis* (Hd=0.72) represents a group with a low genetic diversity (*π*=0.002). The dominant mtDNA genotype among *S. brasiliensis* was haplotype 1 (*n*=42, 51.2%), accounting for the largest proportion of human and animal cases of sporotrichosis in Brazil (Figure 5). The mitochondrial and nuclear data were similar, demonstrating that *S. brasiliensis* was less diverse than *S. schenckii* (Figure 6A). *S. brasiliensis* presented low genetic variation (i.e., a low haplotype and nucleotide diversity) throughout the Brazilian territory, indicating a high level of clonality, and were not geographically restricted to the isolates recovered from the outbreaks in the hyperendemic area of Rio de Janeiro (Figures 6B and 6C).

**Figure 7:**
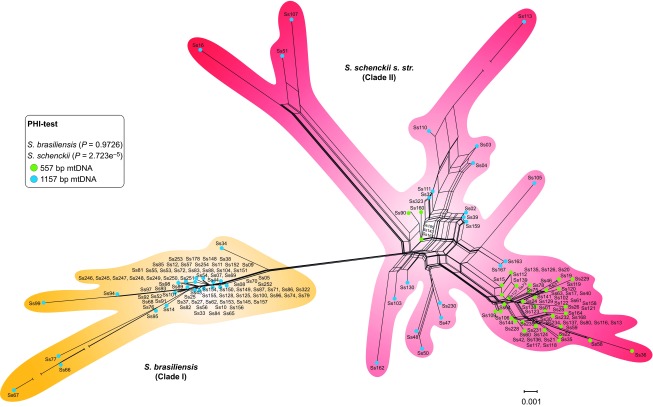
The neighbor network using the uncorrected p-distance among a core set of *Sporothrix* mitochondrial genotypes. Sets of parallel edges (reticulations) in the networks indicate locations of incongruence and potential recombination. Recombination within *S. schenckii s. str.* was also supported by the PHI-test (inbox). Possibilities of recombination were rejected for the *S. brasiliensis* population using NeighborNet and PHI-test analyses, supporting the emergence of a clonal genotype.

NeighborNet, split decomposition and PHI-test analyses were used to detect recombination among clinical and environmental isolates of *Sporothrix*. Evidence of recombination in *S. schenckii s. str*. was observed by large splits/reticulations in NeighborNet and in statistical significance, as determined by the PHI-test (*P*=2.723×10^−5^) (Figure 7). The divergent genotypes of *S. schenckii* revealed strong evidence of recombination inside cluster 2 (mtDNA 1157 bp, *P*=3.64×10^−6^), despite discrete events in cluster 1 (mtDNA 557 bp, *P*=0.1528), as determined using the PHI-test. The clonal expansion of *S. brasiliensis* was confirmed by absent/low recombination events (*P*=0.9726), irrespective of geographical area.

## DISCUSSION

Sporotrichosis has classically been a somewhat obscure disease because it is unique among fungi, as it mainly occurs in the form of outbreaks in endemic areas. However, during the last decade, vast zoonosis has been ongoing in Brazil. The real magnitude of the epidemic is still difficult to establish because sporotrichosis is not an obligatorily reported disease. Our study comprised over 200 cultures from patients living in 14 out of 26 Brazilian states, representing the main endemic areas and clinical forms of the disease. The southern and southeastern parts of the country showed a very high incidence of human cases, which is directly linked to the large epidemic of feline-transmitted sporotrichosis.

Routine diagnostics has become urgent with the introduction of dissimilar species with different types of clinical features and routes of transmission (Supplementary Figure S1). A calmodulin-based phylogeny provided sufficient molecular diversity to identify all pathogenic *Sporothrix* species. The tree (Figure 1) is robust, and its topology corresponds to that of previous studies using the *CAL* locus as a marker.^1,4,5,7^ The geographical distribution and incidence of *S. schenckii s. str.* did not reach epidemic levels, and the isolates formed small but distinct genetic clusters supported by high bootstrap values. The high genetic diversity in the clade of *S. schenckii s. str*. is supported by differences in virulence levels^15^ and chromosomal polymorphisms.^18^

*S. brasiliensis* is by far the most prevalent species in South and Southeast Brazil, with epidemic proportions, as was found in earlier studies.^1,4,5,7^ *S. brasiliensis* was also detected in human hosts in the Central-West and Northeast regions during the years 1997–2004 (Figure 2A), though with a much lower frequency and without a detectable increase in the number of the human cases. The affected patients in those areas did not report traveling into the endemic areas of Rio de Janeiro or Rio Grande do Sul, and an association with diseased cats could not be established.

When the recent outbreaks of feline sporotrichosis^7,13,32,33,34^ are plotted on the areas sampled for human cases (Figure 2B), our data strongly suggest that *S. brasiliensis* is dependent on its feline host for its epidemic emergence in the South and Southeast. The sharp rise in the number of cases in the metropolitan region of Rio de Janeiro since 1998^14^ is likely due to successful zoonotic dispersal by cats to other cats and humans.^7^ The expansion of feline sporotrichosis may have severe implications for the emergence of *S. brasiliensis* in humans. The cat–cat and cat–human transmission in the eco-epidemiology of *S. brasiliensis* is a remarkable characteristic of this pathogen.

In contrast, the classical species *S. schenckii s. str.* showed a more homogeneous distribution throughout the Brazilian territory: it was detected at low frequencies in all regions sampled. Interestingly, *S. schenckii* was the prevalent species among humans in areas free of feline sporotrichosis outbreaks (Figure 2). From an epidemiological and ecological point of view, the low number of cases by *S. schenckii s. str*. suggests that contamination occurs via the classic route, i.e., through contact with plant material. In accordance with this hypothesis, the main occupation of the patients in these areas is related to agricultural practice, especially those involving soil-related activities, gardening and small family farms (Figure 2A; *S. schenckii* cases including soil-related activities in North *n*=3 out of 3–100% Northeast *n*=11 out of 13–84.6% Central-West *n*=6 out of 6–100% Southeast *n*=52 out of 61–85.2% South *n*=16 out of 18–88.8%). In addition, these patients live in neglected areas with poor sanitation and low access to health services, which amplifies the disease risk.

Compared to the alternative route (i.e., via the feline host, as in *S. brasiliensis*^7^), the classic route of infection is expected to be less effective, leading to scattered cases of sporotrichosis in specific occupational patient groups. However, outbreaks are also known to occur by the classic route. The large sapronoses reported from France, the United States, South Africa and China^36,37,38^ indicate that highly specific conditions must be met to promote expansion of the pathogen in plant debris. In the alternative, feline route of transmission, deep scratching is highly effective and a larger number of individuals are at risk of acquiring sporotrichosis.

Diseased cats present a high burden of yeast cells in their lesions.^13,14^ Cat-transmitted cases are occupation independent. In addition, the success of transmission is related to the virulence of the species involved; *S. brasiliensis* has a high degree of pathogenicity in both cat and human hosts.^7,15^ If cats are indeed the prime habitat for the pathogen *S. brasiliensis*, the epidemic is likely to be confined to urban areas that harbor a rich population of susceptible cat hosts. Furthermore, the epidemic is unlikely to end spontaneously, which poses a significant public health problem.

The remarkable situation of the emergence of the apparent primary pathogen, *S. brasiliensis*, among environmental *Sporothrix* species has similarities to other fungal agents with epidemic behavior, such as *Cryptococcus gattii*^39^ or *Cladophialophora carrionii*,^40^ which have opportunistic or environmental sister species, *Cryptococcus neoformans* and *Cladophialophora yegresii*, respectively. An important criterion for true pathogenicity in *S. brasiliensis* is fulfilled through the fact that this dimorphic fungus does not die with the host but contaminates the soil adjacent to its buried host (e.g., cats) and can thus be directly transmitted to the next cat host.^7^ In the zoonotic cycle of sporotrichosis, the feline claws may be the first acquisition site of *Sporothrix* propagules when digging in soil or sharpening on the bark of a tree. Thereafter, the inoculum may be moved into the animal's oral cavity during licking behavior. Therefore, the two main mechanisms of inoculation (i.e., by scratch or bite) are effective in *S. brasiliensis*.

Cross-species pathogen transmission is a driver of sporotrichosis emergence in Brazil. *S. brasiliensis* host predilection, which results in high severe disease in cats, is a striking difference from the epidemics ongoing around the world and in fact has been pivotal to the success of feline outbreaks. A low level of zoonotic transmission exists in other areas;^41,42,43^ however, the causative agent is not *S. brasiliensis*. We demonstrate that the spatial distribution of *S. brasiliensis* is limited to the South and Southeast of Brazil. Therefore, this epidemiological pattern is not homogeneous throughout the Brazilian territory. The frontier expansion of the disease from local to regional appears to be dependent on urban areas with high concentrations of susceptible felines. The presence of *S. brasiliensis* outside Brazil may be regarded as a human and animal threat and raise the risk of a global zoonotic emergence. The epidemiological profile found outside Brazil is usually related to environmental conditions that reflect distinct vector associations, such as soil and decaying wood as well as dissimilar etiological agents, e.g., *S. globosa* in Asia and Europe^35^ and *S. schenckii s. str.* in the United States, Africa and Australia.^6^

*Sporothrix globosa* is rarely involved in sporotrichosis in Brazil, with only four isolates identified with certainty. The species has a worldwide distribution,^4,5,35^ and sporotrichosis caused by *S. globosa* is highly prevalent in Asia,^6^ nearly always without the involvement of feline hosts. The low genetic diversity and its global distribution suggest an association with another environmental source of infection, and its identification is relevant to therapeutic management because the commonly used antifungals, polyenes and azoles, have poor *in vitro* activity against *S. globosa*.^44^

Sporotrichosis is a polymorphic disease.^45^ The balance between the different virulence factors related to the fungus,^46^ as well as the amount and type of inoculum^15^ or the immune status of the host,^2,3^ may contribute to the manifestation of distinct clinical forms. Our epidemiological data (Figure 3) show that *S. brasiliensis* and *S. schenckii s. str.* are able to cause clinical pictures ranging from fixed sporotrichosis with isolated nodules to lymphocutaneous sporotrichosis with lymphatic involvement ascending the limbs (Figure 3). Classically, lymphocutaneous sporotrichosis is the prevalent manifestation among humans.^45^ In the Brazilian epidemic of sporotrichosis, we observed a slight prevalence of fixed cutaneous cases.

A small but increasing number of cases of disseminated sporotrichosis (3.2%) caused by *S. brasiliensis* was noted, but these were not necessarily associated with immunosuppressed patients.^2^ We did not find any correlation between intraspecific genotypes and clinical forms. Our data are consistent with those published by Fernandes *et al.*,^46^ Mesa-Arango *et al.*^47^ and Neyra *et al.*,^48^ who reported that the determinants of clinical forms are related to the patient's immunological system rather than to the genotype of the pathogen.

Host-association appears to have a structuring effect on *Sporothrix* populations.^49^ We found multiple evolutionary and geographic origins in the plant-associated species *S. schenckii s. str*. by evidence from the nuclear and mitochondrial genetic diversity (Figures 4 and 5). In agreement with previous studies,^7^ we observed that *S. brasiliensis*, isolated from cats and humans in outbreak areas, have the same genotype, which confirms the zoonotic transmission of the disease. Epidemics of *S. brasiliensis* often involves familial cases of sporotrichosis, suggesting that several members of the same family become infected by the same animal.^7,50^

Sexual reproduction in fungi has a high impact on infectious outbreaks and the distribution patterns of populations. Recombination plays a critical role in the diversification and evolution of pathogenic species by generating lineages with improved fitness in adverse situations.^51^ However, the event that lies behind the diversification of *S. schenckii s. str.* and the mechanism of the emergence of *S. brasiliensis* are currently unknown.

We found evidence of recombination in *S. schenckii*, but not in *S. brasiliensis*, strongly suggesting that these sister species follow distinct pathways and strategies during epidemics. The reticulated pattern of *S. schenckii* (Figure 7) suggests that recombination among genotypes may have contributed to the evolution of the divergent strains. Although large splits in networks do not necessarily imply recombination, NeighborNet, in conjunction with the PHI-test, can easily detect a recombination signal, as demonstrated in the present study.

Sexual reproduction in *Sporothrix* is likely to occur in an environmental habitat, but the feline outbreak genotypes are prevalently clonal, which does not necessarily imply the absence of sex but does indicate the emergence of a successful genotype. Strictly clonally reproducing pathogens are rare in nature. This epidemic profile is not commonly found in fungal pathogens and, with rare exceptions, true outbreaks occur involving healthy hosts, such as in the case of the outbreak caused by *Cryptococcus gattii* in Vancouver, Canada.^39,52^ This epidemiological pattern of clustered cases and high incidence in a short period of time is better known for viral^53^ and bacterial pathogens;^54^ such events caused by fungi are very rare in humans and are mainly limited to dermatophytes.^55^ The emergence of fungal pathogens in other animals is mostly related to a recent introduction or shift in host, as in the case of white-nose syndrome in bats caused by *Geomyces destructans*,^56^ lethargic crab disease by *Exophiala cancerae*^57^ and the devastation of amphibian populations caused by the chytridiomycete *Batrachochytrium dendrobatidis*.^58,59^

A key factor behind the emergence of the *S. brasiliensis* epidemic is its zoonotic transmission, which distinguishes it as an occupation-independent disease. Our data suggest a habitat shift within *S. brasiliensis* from plant to cat that appears to have occurred in southeastern Brazil. A clonal structure was found in the early expansionary phase of the cat–human epidemic. In contrast, the epidemic, driven by *S. schenckii s. str.*, presented high heterogeneity with a variety of genotypes and diverse virulence profiles. The *S. schenckii* epidemic spreads globally through direct environmental contamination and is usually related to specific occupational patient groups, such as those involved in soil-related activities. This study provides new insights into the spread and epidemiological evolution of sporotrichosis, suggesting that different public health policies and strategies would be required to control future outbreaks.
