# Supplementary material for: Emerging sporotrichosis is driven by clonal and recombinant Sporothrix species
Source: Emerg Microbes Infect. 2014 May 7;3(5):e32–. doi: 10.1038/emi.2014.33 (PMC4051365; doi:10.1038/emi.2014.33)
Supplement: Supplementary Table 1 [file emi201433x1.pdf]

Supplementary Table S1: Strains, species, origin, mtDNA, haplotypes and GenBank accession numbers of *Sporothrix* spp. isolates used in this study.

Isolate code	CBS code	Species	Source	Origin	mtDNA type[a]	Hap[b]	GenBank accession number			Reference[f]
							CAL[c]	mtDNA[d]	ITS[e]	
Ss01	CBS 132961	*S. schenckii*	Feline sporotrichosis	São Paulo, Brazil	557bp	H53	KC693828	KJ020361	-	(1)
Ss02	CBS 132962	*S. schenckii*	Human sporotrichosis	Rio Grande do Sul, Brazil	1157bp	H31	KC693829	KJ020362	-	(1)
Ss03	CBS 132963	*S. schenckii*	Human sporotrichosis	Rio Grande do Sul, Brazil	1157bp	H32	JX077117	KJ020363	KF574446	(2)
Ss04	-	*S. schenckii*	Human sporotrichosis	Rio Grande do Sul, Brazil	1157bp	H32	JX077118	KJ020364	KF961156	(2)
Ss05	CBS 132985	*S. brasiliensis*	Feline sporotrichosis	Minas Gerais, Brazil	1157bp	H01	KC693830	KJ020365	KF961142	(1)
Ss06	CBS 132922	*S. globosa*	Human sporotrichosis	Minas Gerais, Brazil	557bp	H86	JF811336	KJ020366	JN885574	(2)
Ss07	CBS 132986	*S. brasiliensis*	Human sporotrichosis	Minas Gerais, Brazil	1157bp	H01	KC693831	KJ020367	KF961143	(1)
Ss08	-	*S. brasiliensis*	Human sporotrichosis	Minas Gerais, Brazil	1157bp	H01	KC693832	KJ020368	-	(1)
Ss09	-	*S. brasiliensis*	Human sporotrichosis	Minas Gerais, Brazil	1157bp	H01	KC693833	KJ020369	-	(1)
Ss10	CBS 132987	*S. brasiliensis*	Human sporotrichosis	Minas Gerais, Brazil	1157bp	H01	KC693834	KJ020370	-	(1)
Ss11	-	*S. brasiliensis*	Human sporotrichosis	Minas Gerais, Brazil	1157bp	H01	KF943631	KJ020371	-	This study
Ss12	-	*S. brasiliensis*	Human sporotrichosis	Minas Gerais, Brazil	1157bp	H01	KC693835	KJ020372	KF961144	(1)
Ss13	-	*S. schenckii*	Human sporotrichosis	Minas Gerais, Brazil	557bp	H57	KC693836	KJ020373	KF961157	(1)
Ss14	-	*S. brasiliensis*	Human sporotrichosis	Minas Gerais, Brazil	1157bp	H02	KF943632	KJ020374	KF961145	This study
Ss15	-	*S. schenckii*	Human sporotrichosis	Minas Gerais, Brazil	557bp	H69	KC693837	KJ020375	KF961158	(1)
Ss16	-	*S. schenckii*	Human sporotrichosis	Piauí, Brazil	1157bp	H40	JQ041898	KJ020376	KF961159	(3)
Ss17	-	*S. schenckii*	Human sporotrichosis	Paraná, Brazil	557bp	H53	KC693838	KJ020377	KF961160	(1)
Ss19	-	*S. schenckii*	Human sporotrichosis	Paraná, Brazil	557bp	H58	KF943633	KJ020378	KF961161	This study
Ss20	-	*S. schenckii*	Human sporotrichosis	Paraná, Brazil	557bp	H53	JX077119	KJ020379	-	(2)
Ss21	-	*S. schenckii*	Human sporotrichosis	Paraná, Brazil	557bp	H53	JX077120	KJ020380	-	(2)
Ss22	CBS 132964	*S. schenckii*	Human sporotrichosis	Paraná, Brazil	557bp	H73	KF943634	KJ020381	KF961162	This study
Ss24	-	*S. schenckii*	Human sporotrichosis	Paraná, Brazil	557bp	H58	KC693839	KJ020382	-	(1)
Ss25	CBS 132988	*S. brasiliensis*	Human sporotrichosis	Paraná, Brazil	1157bp	H03	KC693840	KJ020383	-	(1)
Ss26	CBS 132965	*S. schenckii*	Human sporotrichosis	Paraná, Brazil	557bp	H77	KC693841	KJ020384	-	(1)
Ss27	-	*S. brasiliensis*	Human sporotrichosis	Paraná, Brazil	1157bp	H04	JX077111	KJ020385	-	(2)
Ss28	-	*S. schenckii*	Human sporotrichosis	Paraná, Brazil	557bp	H58	JX077121	KJ020386	-	(2)
Ss30	-	*S. schenckii*	Human sporotrichosis	Paraná, Brazil	557bp	H72	KF943635	KJ020387	-	This study
Ss31	-	*S. schenckii*	Human sporotrichosis	Paraná, Brazil	557bp	H58	JX077122	KJ020388	-	(2)
Ss32	-	*S. brasiliensis*	Human sporotrichosis	Paraná, Brazil	1157bp	H09	KF943636	KJ020389	-	This study
Ss33	-	*S. brasiliensis*	Human sporotrichosis	Paraná, Brazil	1157bp	H01	KF943637	KJ020390	KF961146	This study

Isolate code	CBS code	Species	Source	Origin	mtDNA type[a]	Hap[b]	GenBank accession number			Reference[f]
							CAL[c]	mtDNA[d]	ITS[e]	
Ss34	-	*S. brasiliensis*	Human sporotrichosis	Paraná, Brazil	1157bp	H44	KF943638	KJ020391	KF961147	This study
Ss35	-	*S. schenckii*	Human sporotrichosis	Paraná, Brazil	557bp	H67	KC693842	KJ020392	-	(1)
Ss36	-	*S. schenckii*	Human sporotrichosis	Paraná, Brazil	557bp	H78	KC693843	KJ020393	KF961163	(1)
Ss37	-	*S. brasiliensis*	Human sporotrichosis	Paraná, Brazil	1157bp	H05	KF943639	KJ020394	KF961148	This study
Ss38	-	*S. brasiliensis*	Human sporotrichosis	Paraná, Brazil	1157bp	H01	KC693844	KJ020395	KF961149	(1)
Ss39	-	*S. schenckii*	Human sporotrichosis	Paraná, Brazil	1157bp	H31	JQ041899	KJ020396	JN885576	(3)
Ss40	-	*S. schenckii*	Human sporotrichosis	Ceará, Brazil	557bp	H79	JQ041900	KJ020397	JN885577	(3)
Ss41	CBS 132923	*S. globosa*	Human sporotrichosis	Ceará, Brazil	557bp	H87	JF811337	KJ020398	KF574456	(2)
Ss42	CBS 132966	*S. schenckii*	Human sporotrichosis	Ceará, Brazil	557bp	H79	KF943640	KJ020399	KF961164	This study
Ss43	-	*S. brasiliensis*	Human sporotrichosis	Ceará, Brazil	1157bp	H03	JX077112	KJ020400	KF961150	(2)
Ss44	-	*S. brasiliensis*	Human sporotrichosis	Ceará, Brazil	1157bp	H01	KF943641	KJ020401	KF961151	This study
Ss45	-	*S. schenckii*	Human sporotrichosis	Goiás, Brazil	-	-	KJ020358	-	KF961165	This study
Ss46	-	*S. schenckii*	Human sporotrichosis	Goiás, Brazil	557bp	H80	KF943642	KJ020402	KF961166	This study
Ss47	-	*S. schenckii*	Human sporotrichosis	Goiás, Brazil	1157bp	H45	JQ041901	KJ020403	KF961118	(3)
Ss48	-	*S. schenckii*	Human sporotrichosis	Goiás, Brazil	1157bp	H46	KF943643	KJ020404	KF961167	This study
Ss49	CBS 132924	*S. globosa*	Human sporotrichosis	Goiás, Brazil	557bp	H88	JF811338	KJ020405	KF961180	(2)
Ss50	-	*S. schenckii*	Human sporotrichosis	Goiás, Brazil	1157bp	H47	KF943644	KJ020406	-	This study
Ss51	-	*S. schenckii*	Human sporotrichosis	Pará, Brazil	1157bp	H48	JQ041902	KJ020407	JN885579	(3)
Ss52	-	*S. brasiliensis*	Human sporotrichosis	São Paulo, Brazil	1157bp	H06	KC693845	KJ020408	KF574444	(1)
Ss53	CBS 132989	*S. brasiliensis*	Feline sporotrichosis	Rio Grande do Sul, Brazil	1157bp	H01	KC693846	KJ020409	-	(1)
Ss54	CBS 132990	*S. brasiliensis*	Feline sporotrichosis	Rio Grande do Sul, Brazil	1157bp	H01	JQ041903	KJ020410	JN885580	(2)
Ss55	-	*S. brasiliensis*	Human sporotrichosis	Rio Grande do Sul, Brazil	1157bp	H01	KC693847	KJ020411	KF961152	(1)
Ss56	-	*S. brasiliensis*	Human sporotrichosis	Rio Grande do Sul, Brazil	1157bp	H01	KC693848	KJ020412	KF961153	(1)
Ss57	-	*S. brasiliensis*	Human sporotrichosis	Rio Grande do Sul, Brazil	1157bp	H01	KF943645	KJ020413	KF961154	This study
Ss58	-	*S. schenckii*	Human sporotrichosis	São Paulo, Brazil	557bp	H81	KF943646	KJ020414	KF961119	This study
Ss59	-	*S. schenckii*	Human sporotrichosis	São Paulo, Brazil	557bp	H55	KF943647	KJ020415	KF961168	This study
Ss60	-	*S. schenckii*	Human sporotrichosis	São Paulo, Brazil	557bp	H82	KF943648	KJ020416	-	This study
Ss61	-	*S. schenckii*	Soil	São Paulo, Brazil	557bp	H83	KF561244	KJ020417	KF574447	(4)
Ss62	CBS 132991	*S. brasiliensis*	Human sporotrichosis	Espírito Santo, Brazil	1157bp	H10	JX077113	KJ020418	KF961120	(2)
Ss63	CBS 132968	*S. schenckii*	Human sporotrichosis	Espírito Santo, Brazil	557bp	H53	JX077123	KJ020419	KF961121	(2)
Ss64	-	*S. schenckii*	Human sporotrichosis	Espírito Santo, Brazil	557bp	H53	JX077124	KJ020420	KF961169	(2)
Ss65	-	*S. brasiliensis*	Human sporotrichosis	Rio de Janeiro, Brazil	1157bp	H01	JX077114	KJ020421	-	(2)

Isolate code	CBS code	Species	Source	Origin	mtDNA type[a]	Hap[b]	GenBank accession number			Reference[f]
							CAL[c]	mtDNA[d]	ITS[e]	
Ss66	-	*S. brasiliensis*	Human sporotrichosis	Rio de Janeiro, Brazil	1157bp	H07	KF943649	KJ020422	-	This study
Ss67	-	*S. brasiliensis*	Human sporotrichosis	Rio de Janeiro, Brazil	1157bp	H08	KF943650	KJ020423	-	This study
Ss68	-	*S. brasiliensis*	Human sporotrichosis	Rio de Janeiro, Brazil	1157bp	H11	JX077115	KJ020424	-	(2)
Ss69	-	*S. brasiliensis*	Human sporotrichosis	Rio de Janeiro, Brazil	1157bp	H12	KC693849	KJ020425	KF961122	(1)
Ss70	-	*S. brasiliensis*	Human sporotrichosis	Rio de Janeiro, Brazil	1157bp	H01	KC693850	KJ020426	-	(1)
Ss71	-	*S. brasiliensis*	Human sporotrichosis	Rio de Janeiro, Brazil	1157bp	H01	KC693851	KJ020427	-	(1)
Ss72	-	*S. brasiliensis*	Human sporotrichosis	Rio de Janeiro, Brazil	1157bp	H01	KC693852	KJ020428	-	(1)
Ss73	-	*S. schenckii*	Human sporotrichosis	Rio de Janeiro, Brazil	557bp	H83	KC693853	KJ020429	KF961170	(1)
Ss74	-	*S. brasiliensis*	Human sporotrichosis	Rio de Janeiro, Brazil	1157bp	H01	KF943651	KJ020430	-	This study
Ss75	-	*S. schenckii*	Human sporotrichosis	Rio de Janeiro, Brazil	557bp	H84	KC693854	KJ020431	KF961123	(1)
Ss76	-	*S. brasiliensis*	Human sporotrichosis	Rio de Janeiro, Brazil	1157bp	H05	KF943652	KJ020432	-	This study
Ss77	-	*S. brasiliensis*	Human sporotrichosis	Rio de Janeiro, Brazil	1157bp	H13	KF943653	KJ020433	-	This study
Ss78	-	*S. schenckii*	Human sporotrichosis	Rio de Janeiro, Brazil	557bp	H85	KC693855	KJ020434	KF961171	(1)
Ss79	-	*S. brasiliensis*	Human sporotrichosis	Rio de Janeiro, Brazil	1157bp	H01	KC693856	KJ020435	-	(1)
Ss80	CBS 132969	*S. schenckii*	Human sporotrichosis	Rio de Janeiro, Brazil	557bp	H68	JX077125	KJ020436	KF961124	(2)
Ss81	-	*S. brasiliensis*	Human sporotrichosis	Rio de Janeiro, Brazil	1157bp	H14	KF943654	KJ020437	-	This study
Ss82	CBS 132992	*S. brasiliensis*	Human sporotrichosis	Rio de Janeiro, Brazil	1157bp	H01	KC693857	KJ020438	KF961125	(1)
Ss83	-	*S. brasiliensis*	Human sporotrichosis	Rio de Janeiro, Brazil	1157bp	H01	KF943655	KJ020439	-	This study
Ss84	-	*S. brasiliensis*	Human sporotrichosis	Rio de Janeiro, Brazil	1157bp	H01	KF943656	KJ020440	-	This study
Ss85	-	*S. brasiliensis*	Human sporotrichosis	Rio de Janeiro, Brazil	1157bp	H01	KF943657	KJ020441	-	This study
Ss86	-	*S. brasiliensis*	Human sporotrichosis	Rio de Janeiro, Brazil	1157bp	H01	KF943658	KJ020442	-	This study
Ss87	CBS 132993	*S. brasiliensis*	Human sporotrichosis	Rio de Janeiro, Brazil	1157bp	H01	KC693858	KJ020443	KF961126	(1)
Ss88	-	*S. brasiliensis*	Human sporotrichosis	Rio de Janeiro, Brazil	1157bp	H01	KF943659	KJ020444	-	This study
Ss89	-	*S. brasiliensis*	Human sporotrichosis	Rio de Janeiro, Brazil	1157bp	H15	KF943660	KJ020445	-	This study
Ss90	-	*S. schenckii*	Human sporotrichosis	Rio de Janeiro, Brazil	557bp	H52	KC693859	KJ020446	-	(1)
Ss91	-	*S. brasiliensis*	Human sporotrichosis	Rio de Janeiro, Brazil	1157bp	H16	KF943661	KJ020447	-	This study
Ss92	-	*S. brasiliensis*	Human sporotrichosis	Rio de Janeiro, Brazil	1157bp	H17	KF943662	KJ020448	-	This study
Ss93	-	*S. brasiliensis*	Human sporotrichosis	Rio de Janeiro, Brazil	1157bp	H18	KF943663	KJ020449	-	This study
Ss94	-	*S. brasiliensis*	Human sporotrichosis	Rio de Janeiro, Brazil	1157bp	H19	KF943664	KJ020450	-	This study
Ss95	-	*S. brasiliensis*	Human sporotrichosis	Rio de Janeiro, Brazil	1157bp	H20	KF943665	KJ020451	-	This study
Ss96	-	*S. brasiliensis*	Human sporotrichosis	Rio de Janeiro, Brazil	1157bp	H01	KF943666	KJ020452	-	This study
Ss97	-	*S. brasiliensis*	Human sporotrichosis	Rio de Janeiro, Brazil	1157bp	H21	KF943667	KJ020453	-	This study

Isolate code	CBS code	Species	Source	Origin	mtDNA type[a]	Hap[b]	GenBank accession number			Reference[f]
							CAL[c]	mtDNA[d]	ITS[e]	
Ss98	-	*S. brasiliensis*	Human sporotrichosis	Rio de Janeiro, Brazil	1157bp	H22	KF943668	KJ020454	-	This study
Ss99	-	*S. brasiliensis*	Human sporotrichosis	Rio de Janeiro, Brazil	1157bp	H23	KF574460	KJ020455	KF574442	(4)
Ss100	-	*S. brasiliensis*	Human sporotrichosis	Rio de Janeiro, Brazil	1157bp	H01	KF943669	KJ020456	-	This study
Ss101	-	*S. brasiliensis*	Human sporotrichosis	São Paulo, Brazil	1157bp	H24	KF943670	KJ020457	-	This study
Ss102	CBS 132970	*S. schenckii*	Human sporotrichosis	São Paulo, Brazil	557bp	H53	KF943671	KJ020458	KF961172	This study
Ss103	-	*S. schenckii*	Human sporotrichosis	São Paulo, Brazil	1157bp	H33	KF943672	KJ020459	-	This study
Ss104	-	*S. brasiliensis*	Human sporotrichosis	Mato Grosso, Brazil	1157bp	H01	KF574461	KJ020460	KF574443	(4)
Ss105	-	*S. schenckii*	Human sporotrichosis	Minas Gerais, Brazil	1157bp	H34	KF943673	KJ020461	KF961127	This study
Ss106	-	*S. schenckii*	Human sporotrichosis	Minas Gerais, Brazil	557bp	H53	KF943674	KJ020462		This study
Ss107	-	*S. schenckii*	Human sporotrichosis	Minas Gerais, Brazil	1157bp	H35	KF943675	KJ020463	KF961128	This study
Ss109	-	*S. schenckii*	Human sporotrichosis	Minas Gerais, Brazil	557bp	H54	KF943676	KJ020464	KF961129	This study
Ss110	-	*S. schenckii*	Human sporotrichosis	Minas Gerais, Brazil	1157bp	H36	KF943677	KJ020465	KF961173	This study
Ss111	CBS 132971	*S. schenckii*	Human sporotrichosis	São Paulo, Brazil	1157bp	H37	KC693860	KJ020466	-	(1)
Ss112	-	*S. schenckii*	Human sporotrichosis	São Paulo, Brazil	557bp	H53	KF943678	KJ020467	-	This study
Ss113	CBS 132972	*S. schenckii*	Human sporotrichosis	São Paulo, Brazil	1157bp	H38	KF943679	KJ020468	KF961130	This study
Ss116	-	*S. schenckii*	Human sporotrichosis	São Paulo, Brazil	557bp	H55	KF943680	KJ020469	KF961131	This study
Ss117	-	*S. schenckii*	Human sporotrichosis	São Paulo, Brazil	557bp	H56	KF943681	KJ020470	-	This study
Ss118	CBS 132974	*S. schenckii*	Human sporotrichosis	São Paulo, Brazil	557bp	H57	JX077126	KJ020471	KF961174	(2)
Ss119	-	*S. schenckii*	Human sporotrichosis	São Paulo, Brazil	557bp	H58	KF943682	KJ020472	KF961175	This study
Ss120	-	*S. schenckii*	Human sporotrichosis	São Paulo, Brazil	557bp	H58	KF943683	KJ020473	-	This study
Ss121	-	*S. schenckii*	Human sporotrichosis	São Paulo, Brazil	557bp	H58	KF943684	KJ020474	-	This study
Ss122	-	*S. schenckii*	Human sporotrichosis	São Paulo, Brazil	557bp	H59	KF943685	KJ020475	KF961132	This study
Ss123	-	*S. schenckii*	Human sporotrichosis	São Paulo, Brazil	557bp	H60	KF943686	KJ020476	KF961133	This study
Ss124	-	*S. schenckii*	Human sporotrichosis	São Paulo, Brazil	557bp	H57	KF943687	KJ020477	KF961176	This study
Ss125	-	*S. brasiliensis*	Human sporotrichosis	São Paulo, Brazil	1157bp	H25	JX077116	KJ020478	-	(2)
Ss126	-	*S. schenckii*	Human sporotrichosis	São Paulo, Brazil	557bp	H53	JQ041904	KJ020479	JN885581	(3)
Ss127	-	*S. schenckii*	Human sporotrichosis	São Paulo, Brazil	557bp	H53	KF943688	KJ020480	-	This study
Ss128	-	*S. brasiliensis*	Human sporotrichosis	São Paulo, Brazil	1157bp	H26	KC693861	KJ020481	KF961155	(1)
Ss129	-	*S. schenckii*	Human sporotrichosis	São Paulo, Brazil	557bp	H61	KF943689	KJ020482	KF961134	This study
Ss130	-	*S. schenckii*	Human sporotrichosis	Pernambuco, Brazil	1157bp	H39	KF943690	KJ020483	KF961135	This study
Ss131	CBS 132926	*S. mexicana*	Human sporotrichosis	Pernambuco, Brazil	-	-	JF811339	-	-	(2)
Ss132	CBS 132927	*S. mexicana*	Human sporotrichosis	São Paulo, Brazil	-	-	JF811340	-	KF574457	(2)

Isolate code	CBS code	Species	Source	Origin	mtDNA type[a]	Hap[b]	GenBank accession number			Reference[f]
							CAL[c]	mtDNA[d]	ITS[e]	
Ss133	CBS 132928	*S. mexicana*	Human sporotrichosis	Pernambuco, Brazil	-	-	JF811341	-	KF961182	(2)
Ss134	-	*S. schenckii*	Human sporotrichosis	Pernambuco, Brazil	-	-	This study	-	-	This study
Ss135	-	*S. schenckii*	Human sporotrichosis	Pernambuco, Brazil	557bp	H53	KF943691	KJ020484	-	This study
Ss136	-	*S. schenckii*	Human sporotrichosis	Pernambuco, Brazil	557bp	H53	KF943692	KJ020485	KF961177	This study
Ss137	-	*S. schenckii*	Human sporotrichosis	Pernambuco, Brazil	557bp	H57	KF574462	KJ020486	KF574448	(4)
Ss138	-	*S. schenckii*	Human sporotrichosis	Paraíba, Brazil	557bp	H62	KF943693	KJ020487	KF961136	This study
Ss139	-	*S. schenckii*	Human sporotrichosis	Paraíba, Brazil	557bp	H63	KF943694	KJ020488	-	This study
Ss140	-	*S. brasiliensis*	Human sporotrichosis	Paraíba, Brazil	-	-	KF574463	-	KF574449	(4)
Ss141	CBS 132975	*S. schenckii*	Human sporotrichosis	Brasília, Brazil	557bp	H58	JQ041905	KJ020489	JN885582	(3)
Ss143	-	*S. schenckii*	Human sporotrichosis	Pará, Brazil	557bp	H50	JQ041906	KJ020490	JN885583	(2)
Ss144	-	*S. schenckii*	Human sporotrichosis	Rio Grande do Sul, Brazil	557bp	H57	KF943695	KJ020491	KF961178	This study
Ss145	-	*S. brasiliensis*	Human sporotrichosis	Rio Grande do Sul, Brazil	1157bp	H01	KF943696	KJ020492	-	This study
Ss148	-	*S. brasiliensis*	Human sporotrichosis	São Paulo, Brazil	1157bp	H01	KF943697	KJ020493	-	This study
Ss149	-	*S. brasiliensis*	Human sporotrichosis	Rio Grande do Sul, Brazil	1157bp	H01	KC693862	KJ020494	-	(1)
Ss150	-	*S. brasiliensis*	Human sporotrichosis	Rio Grande do Sul, Brazil	1157bp	H01	KC693863	KJ020495	-	(1)
Ss151	CBS 132994	*S. brasiliensis*	Canine sporotrichosis	Rio Grande do Sul, Brazil	1157bp	H01	KC693864	KJ020496	-	(1)
Ss152	CBS 132995	*S. brasiliensis*	Feline sporotrichosis	Rio Grande do Sul, Brazil	1157bp	H27	KC693865	KJ020497	-	(1)
Ss153	CBS 132996	*S. brasiliensis*	Feline sporotrichosis	Rio Grande do Sul, Brazil	1157bp	H01	KC693866	KJ020498	-	(1)
Ss154	-	*S. brasiliensis*	Feline sporotrichosis	Rio Grande do Sul, Brazil	1157bp	H01	KC693867	KJ020499	-	(1)
Ss155	-	*S. brasiliensis*	Feline sporotrichosis	Rio Grande do Sul, Brazil	1157bp	H01	KC693868	KJ020500	-	(1)
Ss156	CBS 132997	*S. brasiliensis*	Feline sporotrichosis	Rio Grande do Sul, Brazil	1157bp	H28	KC693869	KJ020501	-	(1)
Ss157	CBS 132998	*S. brasiliensis*	Feline sporotrichosis	Rio Grande do Sul, Brazil	1157bp	H01	KC693870	KJ020502	-	(1)
Ss158	-	*S. schenckii*	Human sporotrichosis	Amazonas, Brazil	557bp	H64	KF943698	KJ020503	KF961137	This study
Ss171	CBS 132999	*S. brasiliensis*	Feline sporotrichosis	Paraná, Brazil	-	-	KC693871	-	-	(1)
Ss172	CBS 133000	*S. brasiliensis*	Feline sporotrichosis	Paraná, Brazil	-	-	KC693872	-	-	(1)
Ss173	CBS 133001	*S. brasiliensis*	Feline sporotrichosis	Paraná, Brazil	-	-	KC693873	-	-	(1)
Ss174	CBS 133002	*S. brasiliensis*	Feline sporotrichosis	Paraná, Brazil	-	-	KC693874	-	-	(1)
Ss188	-	*S. schenckii*	Human sporotrichosis	Pernambuco, Brazil	-	-	KF943699	-	-	This study
Ss189	-	*S. schenckii*	Human sporotrichosis	Pernambuco, Brazil	-	-	KF943700	-	-	This study
Ss190	-	*S. schenckii*	Human sporotrichosis	Pernambuco, Brazil	-	-	KF943701	-	KF961138	This study
Ss192	-	*S. schenckii*	Human sporotrichosis	São Paulo, Brazil	-	-	KJ542523	-	-	This study
Ss193	-	*S. schenckii*	Human sporotrichosis	São Paulo, Brazil	-	-	KJ542524	-	-	This study

Isolate code	CBS code	Species	Source	Origin	mtDNA type[a]	Hap[b]	GenBank accession number			Reference[f]
							CAL[c]	mtDNA[d]	ITS[e]	
Ss194	-	*S. schenckii*	Human sporotrichosis	São Paulo, Brazil	-	-	KJ542525	-	-	This study
Ss195	CBS 132980	*S. schenckii*	Human sporotrichosis	São Paulo, Brazil	-	-	KJ542526	-	-	This study
Ss197	CBS 132981	*S. schenckii*	Human sporotrichosis	São Paulo, Brazil	-	-	KJ542527	-	-	This study
Ss198	-	*S. schenckii*	Human sporotrichosis	São Paulo, Brazil	-	-	KJ542528	-	-	This study
Ss199	-	*S. schenckii*	Human sporotrichosis	São Paulo, Brazil	-	-	KJ542529	-	-	This study
Ss200	CBS 132982	*S. schenckii*	Human sporotrichosis	São Paulo, Brazil	-	-	KJ542530	-	KF961139	This study
Ss201	CBS 132983	*S. schenckii*	Human sporotrichosis	São Paulo, Brazil	-	-	KJ542531	-	-	This study
Ss202	-	*S. schenckii*	Human sporotrichosis	São Paulo, Brazil	-	-	KJ542532	-	-	This study
Ss203	-	*S. schenckii*	Human sporotrichosis	São Paulo, Brazil	-	-	KJ542533	-	-	This study
Ss204	-	*S. schenckii*	Human sporotrichosis	São Paulo, Brazil	-	-	KJ542534	-	-	This study
Ss205	-	*S. schenckii*	Human sporotrichosis	São Paulo, Brazil	-	-	KJ542535	-	-	This study
Ss206	-	*S. schenckii*	Human sporotrichosis	São Paulo, Brazil	-	-	KJ542536	-	-	This study
Ss207	-	*S. schenckii*	Human sporotrichosis	São Paulo, Brazil	-	-	KJ542537	-	-	This study
Ss208	-	*S. schenckii*	Human sporotrichosis	São Paulo, Brazil	-	-	KJ542538	-	-	This study
Ss209	-	*S. schenckii*	Human sporotrichosis	São Paulo, Brazil	-	-	KJ542539	-	KF961140	This study
Ss210	-	*S. schenckii*	Human sporotrichosis	São Paulo, Brazil	-	-	KJ542540	-	-	This study
Ss226	CBS 133003	*S. brasiliensis*	Feline sporotrichosis	São Paulo, Brazil	-	-	KC693875	-	-	(1)
Ss227	CBS 133004	*S. brasiliensis*	Canine sporotrichosis	São Paulo, Brazil	-	-	KC693876	-	-	(1)
Ss236	CBS 132925	*S. globosa*	Human sporotrichosis	Minas Gerais, Brazil	557bp	H89	KC693877	KJ020524	KF961181	(1)
Ss237	-	*S. brasiliensis*	Human sporotrichosis	Espírito Santo, Brazil	-	-	KF943702	-	-	This study
Ss239	-	*S. schenckii*	Human sporotrichosis	São Paulo, Brazil	-	-	KF943703	-	-	This study
Ss240	-	*S. schenckii*	Human sporotrichosis	São Paulo, Brazil	-	-	KF943704	-	KF961141	This study
Ss241	-	*S. schenckii*	Human sporotrichosis	São Paulo, Brazil	-	-	KF943705	-	-	This study
Ss242	-	*S. schenckii*	Human sporotrichosis	Rio Grande do Sul, Brazil	-	-	KF943706	-	-	This study
Ss244	-	*S. brasiliensis*	Human sporotrichosis	Ceará, Brazil	-	-	KF943707	-	-	This study
Ss245	CBS 133005	*S. brasiliensis*	Feline sporotrichosis	Rio de Janeiro, Brazil	1157bp	H29	KC693878	KJ020525	-	(1)
Ss246	CBS 133002	*S. brasiliensis*	Feline sporotrichosis	Rio de Janeiro, Brazil	1157bp	H29	KC693879	KJ020526	-	(1)
Ss247	CBS 133006	*S. brasiliensis*	Feline sporotrichosis	Rio de Janeiro, Brazil	1157bp	H29	KC693880	KJ020527	-	(1)
Ss248	CBS 133007	*S. brasiliensis*	Feline sporotrichosis	Rio de Janeiro, Brazil	1157bp	H29	KC693881	KJ020528	-	(1)
Ss249	CBS 133008	*S. brasiliensis*	Feline sporotrichosis	Rio de Janeiro, Brazil	1157bp	H29	KC693882	KJ020529	-	(1)
Ss250	CBS 133009	*S. brasiliensis*	Feline sporotrichosis	Rio de Janeiro, Brazil	1157bp	H29	KC693883	KJ020530	-	(1)
Ss251	CBS 133010	*S. brasiliensis*	Feline sporotrichosis	Rio de Janeiro, Brazil	1157bp	H29	KC693884	KJ020531	-	(1)

Isolate code	CBS code	Species	Source	Origin	mtDNA type[a]	Hap[b]	GenBank accession number			Reference[f]
							CAL[c]	mtDNA[d]	ITS[e]	
Ss252	CBS 133011	*S. brasiliensis*	Feline sporotrichosis	Rio de Janeiro, Brazil	1157bp	H29	KC693885	KJ020532	-	(1)
Ss253	CBS 133012	*S. brasiliensis*	Feline sporotrichosis	Rio de Janeiro, Brazil	1157bp	H29	KC693886	KJ020533	-	(1)
Ss254	CBS 133013	*S. brasiliensis*	Feline sporotrichosis	Rio de Janeiro, Brazil	1157bp	H29	KC693887	KJ020534	-	(1)
Ss255	CBS 133014	*S. brasiliensis*	Feline sporotrichosis	Rio de Janeiro, Brazil	-	-	KC693888	-	-	(1)
Ss256	CBS 133015	*S. brasiliensis*	Feline sporotrichosis	Rio de Janeiro, Brazil	-	-	KC693889	-	-	(1)
Ss257	CBS 133016	*S. brasiliensis*	Feline sporotrichosis	Rio de Janeiro, Brazil	-	-	KC693890	-	-	(1)
Ss258	CBS 133017	*S. brasiliensis*	Feline sporotrichosis	Rio de Janeiro, Brazil	-	-	KC693891	-	-	(1)
Ss259	CBS 133018	*S. brasiliensis*	Feline sporotrichosis	Rio de Janeiro, Brazil	-	-	KC693892	-	-	(1)
Ss260	CBS 133019	*S. brasiliensis*	Feline sporotrichosis	Rio Grande do Sul, Brazil	-	-	KC693893	-	-	(1)
Ss261	-	*S. brasiliensis*	Human sporotrichosis	Rio Grande do Sul, Brazil	-	-	KC693894	-	-	(1)
Ss265	CBS 133020	*S. brasiliensis*	Human sporotrichosis	Minas Gerais, Brazil	-	-	JN204360	-	KF574445	(5)
ATCC 4823[G]	CBS 132021	*S. brasiliensis*	Feline sporotrichosis	Rio de Janeiro, Brazil	1157bp	H01	KF574459	KJ020535	JQ070114	(4)
IPEC17521	CBS 130107	*S. brasiliensis*	Human sporotrichosis	Rio de Janeiro, Brazil	-	-	AM116874	-	KC113214	(6, 7)
IPEC16503		*S. brasiliensis*	Human sporotrichosis	Rio de Janeiro, Brazil	-	-	AM116875	-	-	(6, 7)
FMR 8337		*S. brasiliensis*	Environmental	Rio de Janeiro, Brazil	-	-	AM116876	-	-	(6, 7)
IPEC16243		*S. brasiliensis*	Human sporotrichosis	Rio de Janeiro, Brazil	-	-	AM116877	-	-	(6, 7)
IPEC17943		*S. brasiliensis*	Human sporotrichosis	Rio de Janeiro, Brazil	-	-	AM116878	-	-	(6, 7)
IPEC22496.5		*S. brasiliensis*	Human sporotrichosis	Rio de Janeiro, Brazil	-	-	AM116879	-	-	(6, 7)
IPEC17331		*S. brasiliensis*	Human sporotrichosis	Rio de Janeiro, Brazil	-	-	AM116880	-	-	(6, 7)
IPEC22542/2		*S. brasiliensis*	Human sporotrichosis	Rio de Janeiro, Brazil	-	-	AM116881	-	-	(6, 7)
IPEC22486		*S. brasiliensis*	Human sporotrichosis	Rio de Janeiro, Brazil	-	-	AM116882	-	-	(6, 7)
IPEC22493.2		*S. brasiliensis*	Human sporotrichosis	Rio de Janeiro, Brazil	-	-	AM116883	-	-	(6, 7)
IPEC17786		*S. brasiliensis*	Human sporotrichosis	Rio de Janeiro, Brazil	-	-	AM116884	-	-	(6, 7)
IPEC16042		*S. brasiliensis*	Human sporotrichosis	Rio de Janeiro, Brazil	-	-	AM116885	-	-	(6, 7)
IPEC15572		*S. brasiliensis*	Human sporotrichosis	Rio de Janeiro, Brazil	-	-	AM116886	-	FN549903	(6, 7)
IPEC17585		*S. brasiliensis*	Human sporotrichosis	Rio de Janeiro, Brazil	-	-	AM116887	-	-	(6, 7)
IPEC17920		*S. brasiliensis*	Human sporotrichosis	Rio de Janeiro, Brazil	-	-	AM116888	-	-	(6, 7)
IPEC16864		*S. brasiliensis*	Human sporotrichosis	Rio de Janeiro, Brazil	-	-	AM116889	-	-	(6, 7)
IPEC17608		*S. brasiliensis*	Human sporotrichosis	Rio de Janeiro, Brazil	-	-	AM116890	-	-	(6, 7)
IPEC22582	CBS 130109	*S. brasiliensis*	Human sporotrichosis	Rio de Janeiro, Brazil	-	-	AM116891	-	KC113213	(6, 7)
IPEC16550		*S. brasiliensis*	Human sporotrichosis	Rio de Janeiro, Brazil	-	-	AM116892	-	-	(6, 7)
IPEC22593		*S. brasiliensis*	Human sporotrichosis	Rio de Janeiro, Brazil	-	-	AM116893	-	-	(6, 7)

Isolate code	CBS code	Species	Source	Origin	mtDNA type[a]	Hap[b]	GenBank accession number			Reference[f]
							CAL[c]	mtDNA[d]	ITS[e]	
IPEC22493.1		*S. brasiliensis*	Human sporotrichosis	Rio de Janeiro, Brazil	-	-	AM116894	-	-	(6, 7)
IPEC22496.4		*S. brasiliensis*	Human sporotrichosis	Rio de Janeiro, Brazil	-	-	AM116895	-	-	(6, 7)
IPEC17307		*S. brasiliensis*	Human sporotrichosis	Rio de Janeiro, Brazil	-	-	AM116896	-	-	(6, 7)
IPEC16456		*S. brasiliensis*	Human sporotrichosis	Rio de Janeiro, Brazil	-	-	AM116897	-	-	(6, 7)
IPEC16919		*S. brasiliensis*	Human sporotrichosis	Rio de Janeiro, Brazil	-	-	AM116898	-	KF574441	(6-8)
IPEC16490[T]		*S. brasiliensis*	Human sporotrichosis	Rio de Janeiro, Brazil	1157bp	H01	AM116899	KJ020512	KF574440	(6-8)
IPEC17692		*S. brasiliensis*	Human sporotrichosis	Rio de Janeiro, Brazil	-	-	AM159127	-	-	(6, 7)
FMR 9034		*S. brasiliensis*	Human sporotrichosis	São Paulo, Brazil	-	-	AM261688	-	-	(6, 7)
FMR 9035		*S. brasiliensis*	Human sporotrichosis	São Paulo, Brazil	-	-	AM261689	-	-	(6, 7)
IPEC28487		*S. brasiliensis*	Human sporotrichosis	Rio de Janeiro, Brazil	-	-	HQ426928	-	-	(9)
IPEC17692		*S. brasiliensis*	Human sporotrichosis	Rio de Janeiro, Brazil	-	-	HQ426929	-	-	(9)
IPEC17786A		*S. brasiliensis*	Human sporotrichosis	Rio de Janeiro, Brazil	-	-	HQ426931	-	-	(9)
IPEC17878		*S. brasiliensis*	Human sporotrichosis	Rio de Janeiro, Brazil	-	-	HQ426932	-	-	(9)
IPEC18782A		*S. brasiliensis*	Human sporotrichosis	Rio de Janeiro, Brazil	-	-	HQ426933	-	-	(9)
IPEC18782B		*S. brasiliensis*	Human sporotrichosis	Rio de Janeiro, Brazil	-	-	HQ426934	-	-	(9)
IPEC25011		*S. brasiliensis*	Human sporotrichosis	Rio de Janeiro, Brazil	-	-	HQ426935	-	-	(9)
IPEC25521		*S. brasiliensis*	Human sporotrichosis	Rio de Janeiro, Brazil	-	-	HQ426936	-	-	(9)
IPEC26611		*S. brasiliensis*	Human sporotrichosis	Rio de Janeiro, Brazil	-	-	HQ426937	-	-	(9)
IPEC26938		*S. brasiliensis*	Human sporotrichosis	Rio de Janeiro, Brazil	-	-	HQ426938	-	-	(9)
IPEC26945		*S. brasiliensis*	Human sporotrichosis	Rio de Janeiro, Brazil	-	-	HQ426939	-	-	(9)
IPEC27022		*S. brasiliensis*	Human sporotrichosis	Rio de Janeiro, Brazil	-	-	HQ426940	-	-	(9)
IPEC27052		*S. brasiliensis*	Human sporotrichosis	Rio de Janeiro, Brazil	-	-	HQ426941	-	-	(9)
IPEC27087		*S. brasiliensis*	Human sporotrichosis	Rio de Janeiro, Brazil	-	-	HQ426942	-	-	(9)
IPEC27130		*S. brasiliensis*	Human sporotrichosis	Rio de Janeiro, Brazil	-	-	HQ426943	-	-	(9)
IPEC27177-2		*S. brasiliensis*	Human sporotrichosis	Rio de Janeiro, Brazil	-	-	HQ426944	-	-	(9)
IPEC27288		*S. brasiliensis*	Human sporotrichosis	Rio de Janeiro, Brazil	-	-	HQ426945	-	-	(9)
IPEC27209		*S. brasiliensis*	Human sporotrichosis	Rio de Janeiro, Brazil	-	-	HQ426946	-	-	(9)
IPEC27372		*S. brasiliensis*	Human sporotrichosis	Rio de Janeiro, Brazil	-	-	HQ426947	-	-	(9)
IPEC27387		*S. brasiliensis*	Human sporotrichosis	Rio de Janeiro, Brazil	-	-	HQ426948	-	-	(9)
IPEC27417		*S. brasiliensis*	Human sporotrichosis	Rio de Janeiro, Brazil	-	-	HQ426949	-	-	(9)
IPEC27445-3		*S. brasiliensis*	Human sporotrichosis	Rio de Janeiro, Brazil	-	-	HQ426950	-	-	(9)
IPEC27930		*S. brasiliensis*	Human sporotrichosis	Rio de Janeiro, Brazil	-	-	HQ426951	-	-	(9)

Isolate code	CBS code	Species	Source	Origin	mtDNA type[a]	Hap[b]	GenBank accession number			Reference[f]
							CAL[c]	mtDNA[d]	ITS[e]	
IPEC34067		*S. brasiliensis*	Human sporotrichosis	Rio de Janeiro, Brazil	-	-	HQ426952	-	-	(9)
IPEC28604		*S. brasiliensis*	Human sporotrichosis	Rio de Janeiro, Brazil	-	-	HQ426953	-	-	(9)
IPEC28701		*S. brasiliensis*	Human sporotrichosis	Rio de Janeiro, Brazil	-	-	HQ426954	-	-	(9)
IPEC28772		*S. brasiliensis*	Human sporotrichosis	Rio de Janeiro, Brazil	-	-	HQ426955	-	-	(9)
IPEC28790		*S. brasiliensis*	Human sporotrichosis	Rio de Janeiro, Brazil	-	-	HQ426956	-	-	(9)
IPEC33605		*S. brasiliensis*	Human sporotrichosis	Rio de Janeiro, Brazil	-	-	HQ426957	-	-	(9)
IPEC33722-1		*S. brasiliensis*	Human sporotrichosis	Rio de Janeiro, Brazil	-	-	HQ426958	-	-	(9)
IPEC34007		*S. brasiliensis*	Human sporotrichosis	Rio de Janeiro, Brazil	-	-	HQ426959	-	-	(9)
IPEC28665		*S. brasiliensis*	Human sporotrichosis	Rio de Janeiro, Brazil	-	-	JN995606	-	-	(10)
IPEC28457		*S. brasiliensis*	Human sporotrichosis	Rio de Janeiro, Brazil	-	-	JN995607	-	-	(10)
IPEC27133		*S. brasiliensis*	Human sporotrichosis	Rio de Janeiro, Brazil	-	-	JN995608	-	-	(10)
IPEC27100		*S. brasiliensis*	Human sporotrichosis	Rio de Janeiro, Brazil	-	-	JN995609	-	-	(10)
IPEC28329		*S. brasiliensis*	Human sporotrichosis	Rio de Janeiro, Brazil	-	-	JN995610	-	-	(10)
ES211		*S. brasiliensis*	Human sporotrichosis	Esp ŕito Santo, Brazil	-	-	JQ915210	-	-	(11)
ES210		*S. brasiliensis*	Human sporotrichosis	Esp ŕito Santo, Brazil	-	-	JQ915211	-	-	(11)
ES213		*S. brasiliensis*	Feline sporotrichosis	Esp ŕito Santo, Brazil	-	-	JQ915212	-	-	(11)
ES212		*S. brasiliensis*	Human sporotrichosis	Esp ŕito Santo, Brazil	-	-	JQ915213	-	-	(11)
IPEC27722		*S. schenckii*	Human sporotrichosis	Rio de Janeiro, Brazil	-	-	HQ426961	-	-	(9)
IOC 1226		*S. schenckii*	Human sporotrichosis	Rio de Janeiro, Brazil	-	-	HQ426962	-	-	(9)
IOC 1113		*S. schenckii*	Human sporotrichosis	Rio de Janeiro, Brazil	-	-	HQ426960	-	-	(9)
26961		*S. schenckii*	Human sporotrichosis	Rio de Janeiro, Brazil	-	-	JN995605	-	-	(10)
IPEC27135		*S. globosa*	Human sporotrichosis	Rio de Janeiro, Brazil	-	-	GU456632	-	-	(12)
SP01	-	*S. schenckii*	Human sporotrichosis	Italy	-	-	-	-	-	(13)
CDM 18	-	*S. schenckii*	Human sporotrichosis	Italy	-	-	-	-	-	(13)
Ss159	CBS 132976	*S. schenckii*	Human sporotrichosis	Japan	1157bp	H42	KF574464	KJ020504	KF574450	(4)
Ss160	-	*S. schenckii*	Human sporotrichosis	Mexico	557bp	H51	KF574465	KJ020505	KF574451	(4)
Ss161	-	*S. schenckii*	Human sporotrichosis	Mexico	557bp	H50	KF574466	KJ020506	KF574452	(4)
Ss162	CBS 132977	*S. schenckii*	Vegetal	Mexico	1157bp	H41	KF574467	KJ020507	KF574453	(4)
Ss163	-	*S. schenckii*	Human sporotrichosis	Peru	1157bp	H43	KF574468	KJ020508	KF574454	(4)
Ss164	-	*S. schenckii*	Human sporotrichosis	Peru	557bp	H70	KF574469	KJ020509	KF574455	(4)
Ss167	CBS 132978	*S. schenckii*	Soil	Peru	1157bp	H43	KF943708	KJ020510	-	This study
Ss168	CBS 132979	*S. schenckii*	Human sporotrichosis	Peru	557bp	H65	KF943709	KJ020511	-	This study

Isolate code	CBS code	Species	Source	Origin	mtDNA type[a]	Hap[b]	GenBank accession number			Reference[f]
							CAL[c]	mtDNA[d]	ITS[e]	
Ss170	-	*S. schenckii*	Human sporotrichosis	Peru	-	-	KF943710	-	-	This study
Ss228	-	*S. schenckii*	Human sporotrichosis	Peru	557bp	H74	KF943711	KJ020517	-	This study
Ss229	-	*S. schenckii*	Human sporotrichosis	Peru	557bp	H66	KF943712	KJ020518	-	This study
Ss230	-	*S. schenckii*	Human sporotrichosis	Peru	1157bp	H49	KF943713	KJ020519	-	This study
Ss231	-	*S. schenckii*	Human sporotrichosis	Peru	557bp	H75	KF943714	KJ020520	-	This study
Ss232	-	*S. schenckii*	Human sporotrichosis	Peru	557bp	H71	KF943715	KJ020521	-	This study
Ss234	-	*S. schenckii*	Human sporotrichosis	Peru	557bp	H57	KF943716	KJ020522	-	This study
Ss235	-	*S. schenckii*	Human sporotrichosis	Peru	557bp	H76	KF943717	KJ020523	-	This study
ATCC 4821[G]	CBS 132984	*S. schenckii*	Human sporotrichosis	USA	557bp	H52	KF574470	KJ020536	JQ070112	(4)
FMR 8600[T]	CBS 120340	*S. globosa*	Human sporotrichosis	Spain	557bp	H89	AM116908	KJ020513	FN549905	(6, 7)
FMR8595	CBS 130104	*S. globosa*	Human sporotrichosis	Spain	557bp	H90	AM116905	KJ020514	-	(6, 7)
ATCC 10268	-	*S. schenckii*	Human sporotrichosis	USA	1157bp	-	AB568599	-	-	(6, 7, 14)
FMR 8598	CBS 130116	*S. globosa*	Human sporotrichosis	Spain	557bp	-	AM116903	KJ020537	KC113226	(6, 7)
KMU 2052		*S. globosa*	Human sporotrichosis	USA	557bp	-	AB568600	-	-	(6, 7, 14)
FMR 9107	CBS 120342	*S. mexicana*	Vegetal	Mexico	-	-	AM398392	-	-	(6, 7)
FMR 9108[T]	CBS 120341	*S. mexicana*	Soil	Mexico	-	-	AM398393	-	-	(6, 7)
FMR 8939[T]	CBS 302.73	*S. pallida*	Soil	United Kingdom	-	-	AM398396	-	-	(6, 7)
FMR 8803		*S. pallida*	Insect	China	-	-	-	-	-	(6, 7)
CBS 359.36[T]	CBS 359.36	*S. schenckii*	Human sporotrichosis	USA	557bp	H50	AM117437	KJ020515	FJ545232	(6, 7)
UTHSC99-173	-	*S. schenckii*	Human sporotrichosis	USA	-	-	-	-	-	(6, 7)
FMR 9280[T]	CBS 937.72	*S. luriei*	Human sporotrichosis	South Africa	1157bp	H30	AM747302	KJ020516	-	(8)
CBS 111110	CBS 111110	*S. pallida*	Insect	Germany	-	-	AM398382	-	-	(6, 7)
CBS 292.55	CBS 292.55	*S. globosa*	Human sporotrichosis	United Kingdom	-	-	AM490354	-	-	(6, 7)
CBS 938.72	CBS 938.72	*S. schenckii*	Human sporotrichosis	France	-	-	AM490340	-	-	(6, 7)
FMR 8604	-	*S. schenckii*	Human sporotrichosis	Peru	-	-	AM117429	-	-	(6, 7)
FMR 8608	-	*S. schenckii*	Human sporotrichosis	Peru	-	-	AM117441	-	-	(6, 7)
FMR 8677	-	*S. schenckii*	Human sporotrichosis	Argentina	-	-	AM117436	-	-	(6, 7)
FMR 8679	-	*S. schenckii*	Human sporotrichosis	Argentina	-	-	AM117445	-	-	(6, 7)
FMR 9023	-	*S. globosa*	Human sporotrichosis	Japan	-	-	AM399016	-	-	(6, 7)
FMR 9051	-	*S. schenckii*	Human sporotrichosis	Venezuela	-	-	AM490342	-	-	(6, 7)
IHEM 3774	-	*S. schenckii*	Human sporotrichosis	Colombia	-	-	AM117447	-	-	(6, 7)
MCCL220085	-	*S. globosa*	Human sporotrichosis	India	-	-	AM490353	-	-	(6, 7)

Isolate code	CBS code	Species	Source	Origin	mtDNA type[a]	Hap[b]	GenBank accession number			Reference[f]
							CAL[c]	mtDNA[d]	ITS[e]	
UTHSC04-1485	-	*S. globosa*	Human sporotrichosis	USA	-	-	AM399015	-	-	(6, 7)
UTHSC04-2235	-	*S. schenckii*	Human sporotrichosis	USA	-	-	AM398984	-	-	(6, 7)
UTHSC05-127	-	*S. globosa*	Human sporotrichosis	USA	-	-	AM398992	-	-	(6, 7)
UTHSC05-2843	-	*S. schenckii*	Human sporotrichosis	USA	-	-	AM399012	-	-	(6, 7)

[a]mtDNA type, based on amplification using the primers 975-8038F and 975-9194R (14)

[b]Haplotype estimated using DnaSP software version 5.10 for the concatenated data set (calmodulin + mtDNA)

[c]Calmodulin data set, amplified and sequenced using the primers CL1 and CL2A (15)

[d]Intergenic region between *atp9* and *cox2* genes in the *Sporothrix* mitochondrial genome, amplified and sequenced using the primers 975-8038F and 975-9194R (14)

[e]Internal transcribed spacer data set including ITS1+5.8S+ITS2, amplified and sequenced using the primers ITS1 and ITS4 (16)

[f]All mtDNA sequences were generated in this study.

IPEC, Instituto de Pesquisa Clínica Evandro Chagas, Fiocruz, Brazil; FMR, Facultat de Medicina i Ciències de la Salut, Reus, Spain; CBS, Centraalbureau voor Schimmelcultures, Utrecht, The Netherlands; KMU, Kanazawa Medical University, Ishikawa, Japan; UTHSC, Fungus Testing Laboratory, University of Texas Health Science Center; MCCL, Mycology Culture Collection Laboratory, Postgraduate Institute of Medical Education and Research, Chandigarh, India; IHEM, BCCM/IHEM Biomedical Fungi and Yeasts Collection, Belgium; ATCC: American Type Culture Collection, Manassas, USA; NK, not known; [T], type strain; [G], genome project. All “Ss” strains belong to the culture collection of Federal University of São Paulo (UNIFESP).

Supplementary References

1. Rodrigues AM, de Melo Teixeira M, de Hoog GS, Schubach TMP, Pereira SA, Fernandes GF, *et al.* Phylogenetic analysis reveals a high prevalence of *Sporothrix brasiliensis* in feline sporotrichosis outbreaks. *PLoS Negl Trop Dis* 2013; **7**(6)**:** e2281.
2. Rodrigues AM, de Hoog S, de Camargo ZP. Emergence of pathogenicity in the *Sporothrix schenckii* complex. *Med Mycol* 2013; **51**(4)**:** 405–412.
3. Fernandes GF, dos Santos PO, Rodrigues AM, Sasaki AA, Burger E, de Camargo ZP. Characterization of virulence profile, protein secretion and immunogenicity of different *Sporothrix schenckii sensu stricto* isolates compared with *S. globosa* and *S. brasiliensis* species. *Virulence* 2013; **4**(3)**:** 241-249.
4. Sasaki AA, Fernandes GF, Rodrigues AM, Lima FM, Marini MM, Feitosa LS*, et al.* Chromosomal polymorphisms in the *Sporothrix schenckii* complex. *PLoS ONE* **9**(1): e86819.
5. Silva-Vergara ML, de Camargo ZP, Silva PF, Abdalla MR, Sgarbieri RN, Rodrigues AM*, et al.* Disseminated *Sporothrix brasiliensis* infection with endocardial and ocular involvement in an HIV-infected patient. *Am J Trop Med Hyg* 2012; **86**(3)**:** 477-480.
6. Marimon R, Cano J, Gené J, Sutton DA, Kawasaki M, Guarro J. *Sporothrix brasiliensis, S. globosa,* and *S. mexicana*, three new *Sporothrix* species of clinical interest. *J Clin Microbiol* 2007; **45**(10)**:** 3198-3206.
7. Marimon R, Gené J, Cano J, Trilles L, Dos Santos Lazéra M, Guarro J. Molecular phylogeny of *Sporothrix schenckii*. *J Clin Microbiol* 2006; **44**(9)**:** 3251-3256.
8. Marimon R, Gené J, Cano J, Guarro J. *Sporothrix luriei*: a rare fungus from clinical origin. *Med Mycol* 2008; **46**(6)**:** 621-625.

9. Oliveira MM, Almeida-Paes R, Muniz MM, Gutierrez-Galhardo MC, Zancope-Oliveira RM. Phenotypic and molecular identification of *Sporothrix* isolates from an epidemic area of sporotrichosis in Brazil. *Mycopathologia* 2011 Oct; **172**(4)**:** 257-267.

10. de Oliveira MME, Sampaio P, Almeida-Paes R, Pais C, Gutierrez-Galhardo MC, Zancope-Oliveira RM. Rapid identification of *Sporothrix* species by T3B fingerprinting. *J Clin Microbiol* 2012; **50**(6)**:** 2159-2162.

11. Oliveira MM, Maifrede SB, Ribeiro MA, Zancope-Oliveira RM. Molecular identification of *Sporothrix* species involved in the first familial outbreak of sporotrichosis in the state of Espirito Santo, Southeastern Brazil. *Mem Inst Oswaldo Cruz* 2013; **108**(7)**:** 936-938.

12. de Oliveira MM, de Almeida-Paes R, de Medeiros Muniz M, de Lima Barros MB, Galhardo MC, Zancope-Oliveira RM. Sporotrichosis caused by *Sporothrix globosa* in Rio De Janeiro, Brazil: case report. *Mycopathologia* 2010; **169**(5)**:** 359-363.

13. Romeo O, Scordino F, Criseo G. New insight into molecular phylogeny and epidemiology of *Sporothrix schenckii* species complex based on calmodulin-encoding gene analysis of Italian isolates. *Mycopathologia* 2011; **172**(3)**:** 179-186.

14. Kawasaki M, Anzawa K, Mochizuki T, Ishizaki H. New strain typing method with *Sporothrix schenckii* using mitochondrial DNA and polymerase chain reaction restriction fragment length polymorphism (PCR–RFLP) technique. *J Dermatol* 2012; **39**(4)**:** 362-365.

15. O'Donnell K, Nirenberg H, Aoki T, Cigelnik E. A multigene phylogeny of the *Gibberella fujikuroi* species complex: Detection of additional phylogenetically distinct species. *Mycoscience* 2000; **41**(1)**:** 61-78.

16. White TJ, Bruns T, Lee S, Taylor J. Amplification and direct sequencing of fungal ribosomal RNA genes for phylogenetics. In: Innis M, Gelfand D, Shinsky J, White T (eds). *PCR Protocols: A Guide to Methods and Applications*. Academic Press1990, pp 315-322.
