# Supplementary material for: Emerging sporotrichosis is driven by clonal and recombinant Sporothrix species
Source: Emerg Microbes Infect. 2014 May 7;3(5):e32–. doi: 10.1038/emi.2014.33 (PMC4051365; doi:10.1038/emi.2014.33)
Supplement: Supplementary Figure S1 [file emi201433x2.pdf]

DNA Barcoding of Brazilian Clinical *Sporothrix* Species

In this study, we thoroughly evaluate the epidemics of sporotrichosis, using molecular tools, which uncover a high level of genetically diverse types. In order to assess the applicability of the ITS as a barcoding marker for the molecular diagnosis of Brazilian clinical *Sporothrix* species, we choose at least one strain from each *CAL* haplotype, and sequenced its ITS region[1], including the internal transcribed spacers 1 and 2 and the 5.8S region, as proposed by Zhou *et al.*[2] In doing so, we improved the diversity of DNA sequences in the clinical clade circulating in the Brazilian territory, and consequently decreased the barcoding-gap.[3] All sequences were deposited online at GenBank (Supplementary Table S1).

Barcoding of *Sporothrix* Species Using the Internal Transcribed Spacer (ITS) Region

The aligned ITS sequences were 634 bp long, corresponding to the ITS1/2 and 5.8S region. The 87 OTUs include 375 invariable characters, 152 variable parsimony-informative sites (23.9 %), and 75 singletons. The clade of pathogenic *Sporothrix* species was well supported with high bootstrap values (84/83) (Supplementary Figure S1). The *S. schenckii s. str.* were scattered throughout the two previously detected groups[2], with a higher concentration in the Latin American group, opposite to group that harbors the type strain of *S. schenckii* (CBS 356.36). All clinical isolates were correctly identified using the full length ITS1/2 and 5.8S region, matching 100 % of the calmodulin identification, thus confirming the utility of this marker for molecular diagnosis of clinical specimens.[2]

In a genetically diverse population, the use of a marker that reduces the barcoding gap may increase the reliability and efficiency of the pathogen identification.[2,3] According

to Zhou *et al.*[2], the ITS region can be used as a barcode marker for the identification of clinical *Sporothrix* species; this region (ITS1/2 + 5.8S) clearly differentiated the Brazilian specimens in our study (Supplementary Figure S1). Thus, due to the ease of amplification and sequencing of the ITS region, it would be very useful to choose this full region over the calmodulin marker for the molecular diagnosis of sporotrichosis, whereas calmodulin remains a great marker for studies on genetic diversity and evolution.

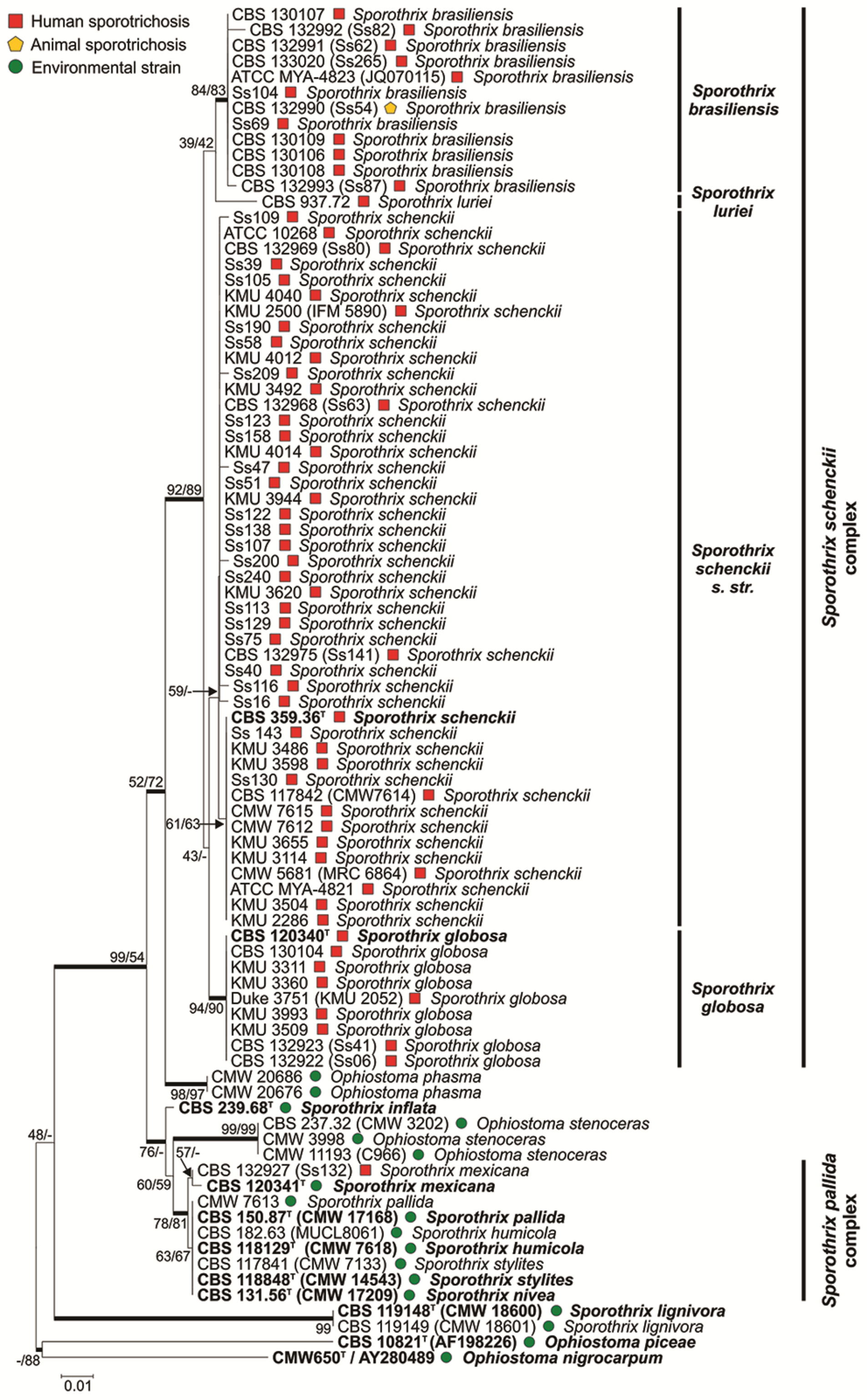

Supplementary Figure S1: Barcoding of clinical *Sporothrix* species, using the ITS1/2 + 5.8S region as a marker. Phylogenetic relationships, inferred from maximum likelihood based on 87 sequences. Numbers close to the branches represent indices of support (NJ/ML), based on 1,000 bootstrap replications. Branches with bootstrap support higher than 70% are indicated in bold.

Supplementary Reference

1. White TJ, Bruns T, Lee S, Taylor J. Amplification and direct sequencing of fungal ribosomal RNA genes for phylogenetics. In: Innis M, Gelfand D, Shinsky J, White T (eds). *PCR Protocols: A Guide to Methods and Applications*. Academic Press1990, pp 315-322.
2. Zhou X, Rodrigues AM, Feng P, Hoog GS. Global ITS diversity in the *Sporothrix schenckii* complex. *Fungal Divers* 2013**:** 1-13.
3. Meyer CP, Paulay G. DNA Barcoding: Error rates based on comprehensive sampling. *PLoS Biol* 2005; **3:** e422.
